# Sex Modifies the Association Between Planetary Health Diet Adherence and Kidney Function Decline in US Adults Aged 50 Years and Older

**DOI:** 10.3390/nu18172850

**Published:** 2026-09-01

**Authors:** Guido Gembillo, Luca Soraci, Alberto La Spada, Paolo Fabbietti, Claudia Lo Re, Maria Federica Ricca, Andrea Corsonello, Domenico Santoro

**Affiliations:** 1 Unit of Nephrology and Dialysis, Department of Clinical and Experimental Medicine, University of Messina, Via Consolare Valeria n 1, 98125 Messina, Italy; guidogembillo@live.it (G.G.); laspada.alberto@gmail.com (A.L.S.); loreclaudia96@gmail.com (C.L.R.); mariafedericaricca8@gmail.com (M.F.R.); 2 Unit of Geriatric Medicine and Clinical Epidemiology, Italian National Research Center on Aging (IRCCS INRCA), Cda Muoio Piccolo, 87100 Cosenza, Italy; l.soraci@inrca.it (L.S.); p.fabbietti@inrca.it (P.F.); a.corsonello@inrca.it (A.C.); 3 Department of Pharmacy, Health and Nutritional Sciences, University of Calabria, 87036 Rende, Italy

**Keywords:** planetary health diet, cystatin C, estimated glomerular filtration rate, chronic kidney disease, sex differences, dietary patterns, kidney function decline, nutritional epidemiology, older adults

## Abstract

**Background/Objectives:** Diet is a modifiable determinant of kidney health, and sex differences in kidney physiology support sex-specific evaluation of dietary patterns. We examined the association of Planetary Health Diet Index (PHDI) adherence with cystatin C-based eGFR decline, and its modification by sex, in US adults aged 50 years and older. **Methods:** We analyzed 2126 Health and Retirement Study participants (950 men, 1176 women) with diet assessed by food frequency questionnaire in 2013 and cystatin C-based eGFR measured in 2012 and 2016. Survey-weighted regression models, adjusted for baseline eGFR and for demographic, socioeconomic, lifestyle and clinical covariates, related PHDI to eGFR change and to a ≥30% relative eGFR decline; sex interaction was tested on the multiplicative and additive scales. **Results:** PHDI adherence was nonsignificantly associated with lower eGFR decline (β per 10-point increment 0.218; 95% CI −0.065 to 0.466). Among women, each 10-point-higher score was associated with slower eGFR decline (β 0.355; 95% CI 0.029 to 0.681; *p* = 0.034) and 20% lower odds of ≥30% eGFR decline (OR 0.799; 95% CI 0.687 to 0.930; *p* = 0.005); no significant association was observed in men (β 0.080; 95% CI −0.294 to 0.454; OR 1.080; 95% CI 0.901 to 1.295). Sex modified the association with ≥30% eGFR decline on both a multiplicative (*p* = 0.002) and additive scale (*p* < 0.001). **Conclusions:** PHDI adherence was associated with slower kidney function decline in women aged 50 years and older, with non-significant effect in men. Sex merits consideration as a biological modifier when plant-forward diets are evaluated as potentially nephroprotective strategies.

## 1. Introduction

Kidney function declines with age, and in older adults a faster rate of decline predicts higher all-cause and cardiovascular mortality [1]. Diet is considered one of the potentially modifiable determinants of this trajectory, and the Planetary Health Diet, proposed by the EAT-Lancet Commission, describes an eating pattern rich in plant protein and whole grains and limited in red and processed meat [2]. Adherence has been associated with lower mortality and cardiovascular disease in this same cohort of US adults aged 50 years and older [3], and in two cross-sectional analyses of NHANES we related it to kidney health and body-mass distribution [4] and to medication burden in older adults with chronic kidney disease (CKD) [5]. Studying kidney function decline in this age group is complicated by its measurement: serum creatinine reflects skeletal muscle in addition to glomerular filtration, and age-related loss of muscle mass has been associated with overestimation of repeatedly measured creatinine-based eGFR, potentially attenuating the apparent rate of kidney function decline [6].

Cystatin C is less affected by muscle mass than creatinine [6,7]. Current guidelines recommend incorporating cystatin C into GFR estimation when available because it improves accuracy and risk stratification [8,9]. We therefore evaluated kidney function decline using cystatin C-based eGFR, while acknowledging that cystatin C is also influenced by adiposity and inflammation [7,10].

Adherence to the Planetary Health Diet has been prospectively associated with a lower risk of CKD across several cohorts. In the UK Biobank, the highest adherence quintile was associated with a hazard ratio of 0.74 (95% CI 0.65–0.85) compared with the lowest [11], and inverse associations have been reported using different Planetary Health Diet scores in further analyses of the UK Biobank and of a Chinese prospective cohort [12,13]. In two prospective cohorts, greater adherence was associated with lower incident CKD among women aged 40 years and older, without a clear association in men [12]. However, these studies share both an endpoint and a marker. The endpoint is incident CKD, reflecting the crossing of a diagnostic threshold rather than the rate of kidney function decline; in one cohort, longitudinal eGFR measurements were unavailable for most participants and CKD ascertainment relied on administrative codes [11,13]. Moreover, kidney function was assessed using serum creatinine, whose dependence on muscle mass may be particularly relevant in older adults.

Sex differences in kidney health have received increasing attention, with calls to evaluate kidney outcomes separately in women and men [14]. Men are more likely than women to progress to kidney failure despite a higher prevalence of CKD stages G3–G5 among women [15], and population-based measured-GFR data show a slower, more nearly linear age-related decline in women compared with a steeper, curvilinear decline in men [14]. Cystatin C-based eGFR and albuminuria are stronger predictors of adverse outcomes in women than in men [14], supporting the importance of evaluating cystatin C-based kidney outcomes separately by sex. Studies examining dietary patterns and kidney function decline with attention to sex have largely been restricted to women [16]. Previous studies have suggested that dietary patterns associated with kidney outcomes may differ between women and men, supporting the need for sex-specific evaluation of nutritional strategies [17]. Plant-based dietary indices have been evaluated only in mixed-sex cohorts using creatinine-based kidney function measures [18]. Furthermore, determinants of creatinine- and cystatin C-based eGFR differ between women and men, with dietary protein intake contributing to creatinine–cystatin discordance in men [19]. The association between adherence to the Planetary Health Diet and the rate of cystatin C-based eGFR decline, and its variation between women and men, has not, to our knowledge, been examined. To establish whether the findings were consistent across filtration markers, we additionally examined the cross-sectional association between PHDI and kidney function estimated with the combined 2021 CKD-EPI creatinine–cystatin C equation.

The Health and Retirement Study is a nationally representative cohort of US adults aged 50 years and older [20] that assessed diet using a validated Harvard food frequency questionnaire in 2013 and measured cystatin C from dried blood spots [21], providing repeated estimates of kidney function. Using this cohort, we examined whether adherence to the Planetary Health Diet was associated with the rate of cystatin C-based eGFR decline and whether this association differed by sex.

## 2. Materials and Methods

This study used data from the Health and Retirement Study (HRS), a nationally representative longitudinal cohort of community-dwelling US adults aged 50 years and older [20]. Dietary data were obtained from the 2013 Health Care and Nutrition Study (HCNS), an HRS sub-study administering a validated Harvard food frequency questionnaire to a random subsample of HRS participants. Kidney function was assessed from cystatin C measured from dried blood spots in the HRS biomarker sub-study.

**Figure 1:**
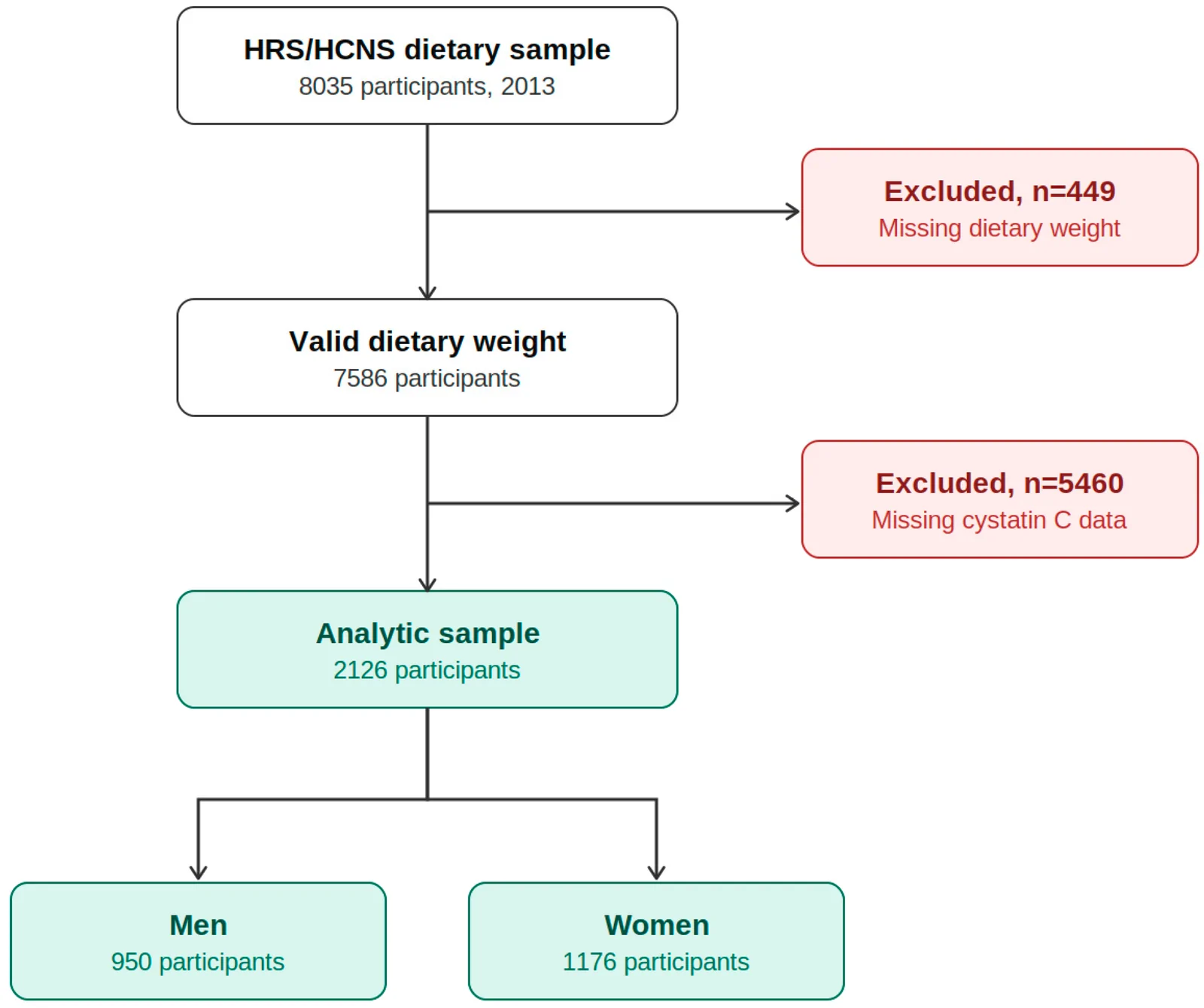
Flow chart illustrating the derivation of the analytic sample, from the initial dietary cohort to the final study population stratified by sex.

The initial sample comprised 8035 participants with complete dietary data. Of these, 7586 (94.4%) had a valid dietary survey weight and complex-design variables (strata, primary sampling units) required for survey-weighted analysis; the present sample was further restricted to the 2126 participants with a cystatin C measurement at both the 2012 and 2016 waves (Figure 1).

Compared with excluded participants, those included were slightly younger (64.3 vs. 66.3 years; *p* < 0.001), although the standardized mean difference was modest (SMD = 0.200), and had a lower prevalence of hypertension (52.1% vs. 55.8%; *p* = 0.019; SMD = 0.076). No other characteristic differed significantly between the two groups, including race/ethnicity, education, BMI, smoking, alcohol use, diabetes, cardiovascular disease, stroke, cancer, chronic lung disease, depressive symptoms, or PHDI score (all *p* > 0.05; all SMD < 0.07).

Data access was granted through the Health and Retirement Study restricted data portal following execution of a Data Use Agreement with the University of Michigan. All analyses were performed on de-identified data in compliance with the terms of that agreement. The Health and Retirement Study is approved by the University of Michigan Institutional Review Board and all participants provided written informed consent to the original study. The present analysis is a secondary use of de-identified data and was therefore exempt from additional ethical review.

### 2.1. Dietary Exposure

Dietary intake was assessed with the semi-quantitative Harvard food frequency questionnaire (FFQ) administered in the 2013 HCNS. For each food item the questionnaire specifies a natural portion size and asks participants to report their usual consumption frequency over the preceding 12 months on a nine-category scale, ranging from “never or less than once per month” to “six or more times per day”. Completed questionnaires were processed centrally by the Harvard T.H. Chan School of Public Health Nutrition Department, which released the HCNS nutrient total database used in the present analysis: a participant-level file reporting usual intake of each food group in servings per day together with total energy intake and 215 additional nutrient variables, derived by linking item frequencies to the Harvard nutrient composition database. This file incorporates contributions from all foods and mixed dishes; composite items were assigned to a single predominant PHDI component based on the Harvard nutrient composition database (e.g., “beef, pork, or lamb sandwich or mixed dish” assigned to red/processed meat; “chicken or poultry, frozen dinner”, assigned to poultry), consistent with the original design of the FFQ [22]; indeed, such items are intended to capture intake of a single predominant food group rather than secondary ingredients assessed by other items. This file was used as the source of all dietary variables in the present analysis.

Construction of the exposure proceeded in three steps, detailed in Supplementary Table S1. First, food group intakes were extracted from the HCNS nutrient total database in servings per day and converted to grams per day by multiplying by the standard gram weight of one serving of the corresponding food group as specified in the USDA Food and Nutrient Database for Dietary Studies [23]; second, gram-per-day intakes of individual FFQ items were summed within each PHDI component; most components, including non-starchy vegetables, fruit, legumes, soy, dairy, red and processed meat, poultry, eggs, fish and shellfish, and starchy vegetables, were constructed by summing gram-per-day intakes across multiple FFQ items; by contrast, whole grains, saturated and unsaturated fats were derived directly from pre-aggregated HCNS nutrient-total variables already expressed in grams; similarly, added sugar was derived from a pre-aggregated variable combining added sugar and fruit juice sugar. Third, absolute daily gram intakes were scored against the EAT–Lancet Commission reference diet [2] using the dietaryindex R package [24], which assigns each of the 15 components a score proportional to its distance from the reference intake, for a maximum total of 140 points, with soy and non-soy legumes weighted at half and the remaining components weighted fully; higher scores denote greater adherence to a plant-rich, sustainable dietary pattern. Added fats and added sugar were expressed as a percentage of total energy intake, as required by the scoring algorithm (Supplementary Table S1). Total energy intake was winsorized to sex-specific bounds (800–4200 kcal/day in men; 500–3500 kcal/day in women), retaining all 568 affected participants (7.1% of respondents with complete dietary data).

For descriptive purposes, food group intakes were additionally reported standardized per 1000 kcal/day.

### 2.2. Kidney Function Assessment

Kidney function was estimated from serum cystatin C measured at 2012 (baseline) and 2016 (follow-up) waves. This biomarker was measured from dried blood spots (DBSs) in 2012 and, in 2016, from both DBSs and venous blood samples (VBSs), depending on participant consent and completion [21,25]. DBS-based cystatin C was assayed via immunoturbidimetric assay at HRS laboratories; DBS cards were collected via finger stick, air-dried, and shipped to the processing laboratory [21]. VBS-based cystatin C, available for a subsample of participants in the 2016 Venous Blood Study, was assayed via standard clinical immunoassay at a Clinical Laboratory Improvement Amendments-certified laboratory, by using the Roche COBAS 6000 Chemistry analyser [25]; the laboratory inter-assay CV was 4.3% at a concentration of 0.75 mg/L and 3.2% at a concentration of 3.83 mg/L [25]. Further details on assay laboratories, calibration procedures, and cross-wave comparability were previously reported [21,25].

DBS values were not used in their raw form: HRS provides regression-based equations calibrating each DBS assay wave to the corresponding NHANES serum assay scale (NHANES 1999–2002 being the reference for cystatin C at every DBS wave); all DBS values were also converted into venous blood equivalents to facilitate longitudinal analysis, as previously reported [21]; the conversion makes cystatin C equivalent to serum values [21]. Among participants with both 2016 measurements available, agreement between the calibrated DBS and VBS values was good (Pearson’s r = 0.87; mean difference −0.03 mg/L).

The eGFR was calculated using the 2021 CKD-EPI cystatin C-only equation [26], which does not include a race coefficient and yields eGFR in mL/min/1.73 m^2^:

eGFR (mL/min/1.73 m^2^) = 133 × min(cystatin C/0.8, 1)^−0.499^ × max(cystatin C/0.8, 1)^−1.328^ × 0.996^age^ × 0.932 (if female).

Two outcomes were defined. The primary outcome was the annualized change in cystatin C-based eGFR, computed as (eGFR_2016_ − eGFR_2012_) divided by the individual follow-up interval in years and expressed in mL/min/1.73 m^2^ per year, with negative values denoting decline. The secondary outcome was rapid progression, defined as a relative decline of at least 30% in eGFR between 2012 and 2016, a threshold recognized as a surrogate endpoint for progression to kidney failure.

As a sensitivity analysis, kidney function at the 2016 wave was also estimated with the 2021 CKD-EPI combined creatinine–cystatin C equation, which incorporates venous serum creatinine and cystatin C and does not include a race coefficient [26]. Serum creatinine was measured in serum by the Roche enzymatic method (Roche Diagnostics, Indianapolis, IN, USA) on a Roche COBAS 6000 Chemistry Analyzer. The method was calibrated using a National Institute of Standards and Technology standard traceable to a reference material SRM 909 b (Isotope Dilution Mass Spectroscopy); the laboratory coefficient of variation was 2.9% at a concentration of 0.895 mg/dL and 2.8% at a concentration of 3.93 mg/dL, as previously reported [25].

Because venous creatinine was available only at the 2016 wave, this combined-marker analysis was necessarily cross-sectional and could not be extended to the longitudinal rate of decline.

### 2.3. Covariates

Covariates were selected a priori based on established or plausible associations with dietary intake, kidney function, or both, and included age, sex, race/ethnicity, years of education (years), and the poverty–income ratio, as demographic and socioeconomic indicators; baseline eGFR, body mass index (BMI), current smoking, alcohol consumption, moderate-to-vigorous physical activity at least once weekly, hypertension, type 2 diabetes, cardiovascular disease, stroke, cancer, chronic lung disease and depressive symptoms (based on the CES-D score), number of annual medical visits as a proxy for healthcare utilization, number of medications and medications used for heart disease, hypertension, or diabetes. More in detail, age (years) and sex were obtained from the 2012 baseline interview. Race/ethnicity was derived by combining self-reported race and Hispanic origin into four mutually exclusive categories (Non-Hispanic White, Non-Hispanic Black, Hispanic, Non-Hispanic other race/ethnicity). Education was measured as self-reported years of completed schooling; socioeconomic status was additionally captured using the household poverty–income ratio, calculated by HRS as the ratio of household income to the federal poverty threshold for household size and composition. Body mass index (BMI, kg/m^2^) was calculated by HRS from self-reported or measured height and weight; current smoking status was defined as an affirmative response to currently smoking cigarettes; alcohol consumption was measured as the self-reported number of days per week on which the participant drank any alcoholic beverage; moderate-to-vigorous physical activity was defined as engaging in vigorous activities (e.g., aerobics, running, swimming) and/or moderate activities (e.g., gardening, walking) at a frequency of at least once weekly, based on the corresponding HRS activity-frequency items, versus less frequent or no activity.

Hypertension, type 2 diabetes, cardiovascular disease, stroke, cancer, and chronic lung disease were each defined as an affirmative response to the HRS question of whether a doctor had ever told the participant they had that condition. Depressive symptoms were assessed using the 8-item CES-D score, with higher scores indicating greater symptom burden. Healthcare utilization was captured as the self-reported number of doctor visits in the past two years. Medication use and number of drugs was assessed from the 2012 HRS core survey [20].

### 2.4. Statistical Analysis

Baseline characteristics were described using survey-weighted means and standard deviations or median and interquartile ranges for continuous variables and survey-weighted frequencies and percentages for categorical variables. Differences between men and women were assessed using survey-weighted linear regression and survey-weighted rank-based tests for continuous variables depending on their distribution and the Rao-Scott chi-square test for categorical variables, both accounting for the complex sampling design of the HRS. Mean daily food group intakes were estimated using survey-weighted means overall and stratified by sex, with sex differences tested using survey-weighted linear regression. Differences in food group intake across PHDI quartiles were assessed using survey-weighted linear regression of each food group on PHDI quartile as a continuous (ordinal trend) variable, with *p*-trend values reported.

All primary analyses accounted for the complex survey design of the HRS incorporating sampling weights (HCNS dietary weights), primary sampling units (SECU), and strata, implemented using the survey package in R (version 4.3.2; R Foundation for Statistical Computing, Vienna, Austria).

### 2.5. Primary Outcome

Survey-weighted linear regression models examined the association between PHDI and annualized eGFR change as the primary outcome. PHDI was modeled in three ways: as a continuous variable per 10-point increment, as quartiles with Q1 as the reference category, and as an ordinal variable across quartiles to assess linear trend. Quartile boundaries were defined using the weighted distribution of PHDI scores in the analytic sample.

All regression models were adjusted for the following pre-specified covariates: age, sex, race/ethnicity, poverty–income ratio, education years, baseline eGFR, BMI, moderate-to-vigorous physical activity, current smoking, alcohol consumption, chronic diseases (hypertension, type 2 diabetes, cardiovascular disease, chronic lung disease, stroke, cancer, and CES-D score), number of annual medical visits, number of drugs and specific medication classes used for hypertension, diabetes, and heart diseases.

Regression coefficients (β) with 95% confidence intervals (CI) and two-sided *p*-values are reported. A positive β coefficient indicates less eGFR decline.

### 2.6. Secondary Outcome

Survey-weighted logistic regression related PHDI to the odds of a ≥30% decline in eGFR, using the same three parameterizations of PHDI and the same covariate set. Odds ratios with 95% confidence intervals were reported.

### 2.7. Effect Modification by Sex

All models were fitted in the overall sample and separately in men and women; sex-stratified models included the already specified covariates except for sex. Effect modification was additionally tested formally in the pooled sample. On the multiplicative scale, a PHDI × sex product term was added to the model and the corresponding *p*-value for interaction was reported. On the additive scale, we estimated the average marginal effect of a 10-point-higher PHDI on the probability of a ≥30% decline within each sex by marginal standardization over the observed covariate distribution, obtaining sex-specific absolute risk differences; the difference between these two risk differences, with its 95% confidence interval and *p*-value (delta method applied to the survey-linearized covariance matrix), quantifies additive interaction.

Statistical significance was defined as *p* < 0.05. All analyses were performed in R (version 4.3.2; R Foundation for Statistical Computing, Vienna, Austria) using the survey and marginaleffects packages.

### 2.8. Sensitivity Analyses

Sensitivity analyses were conducted in the subsample of 1698 participants with available creatinine and cystatin C assessment at the 2016 visit. The association between PHDI and this combined-marker eGFR was examined cross-sectionally, using the same survey-weighted linear regression approach and covariate set with the exception of baseline eGFR, given the cross-sectional nature of the analysis; PHDI was modeled (per 10-point increment), by quartile, and as a linear trend across quartiles, overall and stratified by sex, with formal testing of PHDI-by-sex interaction on the multiplicative scale.

Furthermore, to address the possibility that dietary reporting in 2013 was influenced by early, subclinical decline in kidney function already present at the 2012 baseline, all primary models were re-fitted in the subgroup of 1453 participants without biochemical evidence of reduced kidney function at baseline (eGFR_2012_ ≥ 60 mL/min/1.73 m^2^).

## 3. Results

### 3.1. Baseline Characteristics of the Analytic Sample

The analytic sample comprised 2126 participants with a mean age of 64.3 ± 9.2 years, of whom 55.3% were women and 79.2% were Non-Hispanic White (Table 1).

Compared with women, men consumed alcohol more frequently (1.74 vs. 1.04 days/week; *p* < 0.001), were more likely to engage in moderate-to-vigorous physical activity (82.2% vs. 71.5%; *p* < 0.001), and had a higher prevalence of cardiovascular disease (21.7% vs. 17.4%; *p* = 0.011) and stroke (7.3% vs. 4.7%; *p* = 0.045). Current smoking and type 2 diabetes were also more frequent among men, although these differences were not statistically significant (15.0% vs. 12.5%, *p* = 0.107; and 20.3% vs. 17.0%, *p* = 0.092, respectively). Women had higher depressive symptom scores than men (1.4 vs. 1.2; *p* = 0.019) and lower baseline eGFR (72.0 vs. 76.3 mL/min/1.73 m^2^; *p* = 0.004). At baseline, 68.4% of participants had an eGFR ≥ 60 mL/min/1.73 m^2^, while 18.4%, 9.9% and 3.3% had eGFR 45–59.9, 30–44.9 and <30 mL/min/1.73 m^2^, respectively; the distribution across these categories differed by sex, with a higher prevalence of reduced eGFR among women than men (34.6% vs. 27.9% with eGFR < 60; *p* = 0.005). The mean PHDI score was significantly higher among women than men (78.3 ± 12.1 vs. 75.9 ± 12.3 points; *p* < 0.001), indicating slightly greater adherence to the planetary health diet among women.

### 3.2. Food Group Intake Overall, by Sex and Across PHDI Quartiles

**Figure 2:**
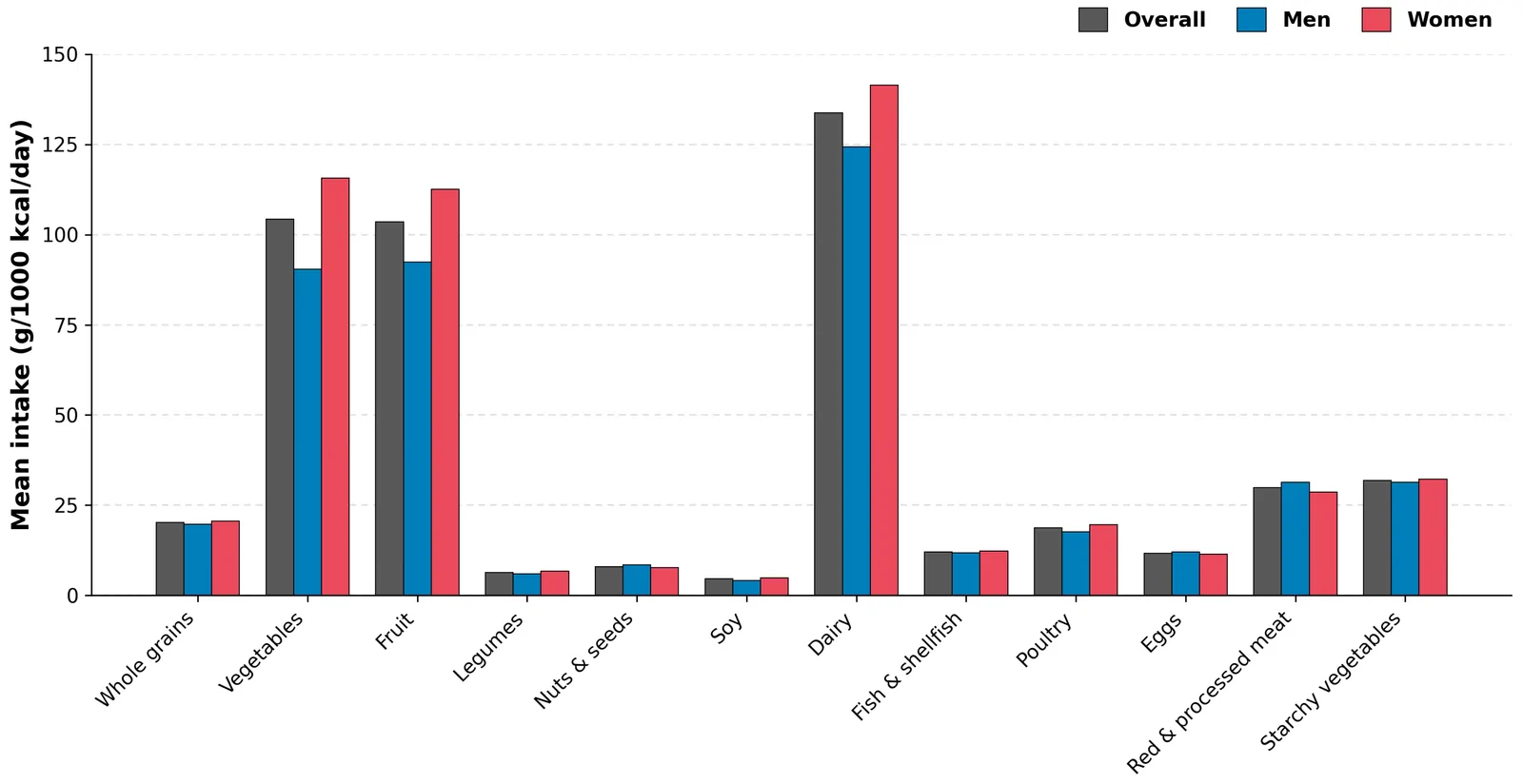
Survey-weighted mean (SD) standardized daily intake (g/1000 kcal/day) of Planetary Health Diet Index (PHDI) food components overall and by sex.

Survey-weighted mean daily standardized food group intakes are presented in Table 2 and Figure 2.

Women consumed significantly more vegetables (115.6 vs. 90.4 g/1000 kcal), fruit (112.6 vs. 92.4 g/1000 kcal), dairy (141.4 vs. 124.4 g/1000 kcal), and poultry (19.6 vs. 17.6 g/1000 kcal) than men. In contrast, men consumed significantly more red and processed meat than women (31.3 vs. 28.6 g/1000 kcal). No significant sex differences were observed for whole grains, legumes, nuts and seeds, soy, fish and shellfish, eggs, or starchy vegetables. Added unsaturated fat, added saturated and trans fat, and added sugar intake also did not differ significantly between men and women.

**Figure 3:**
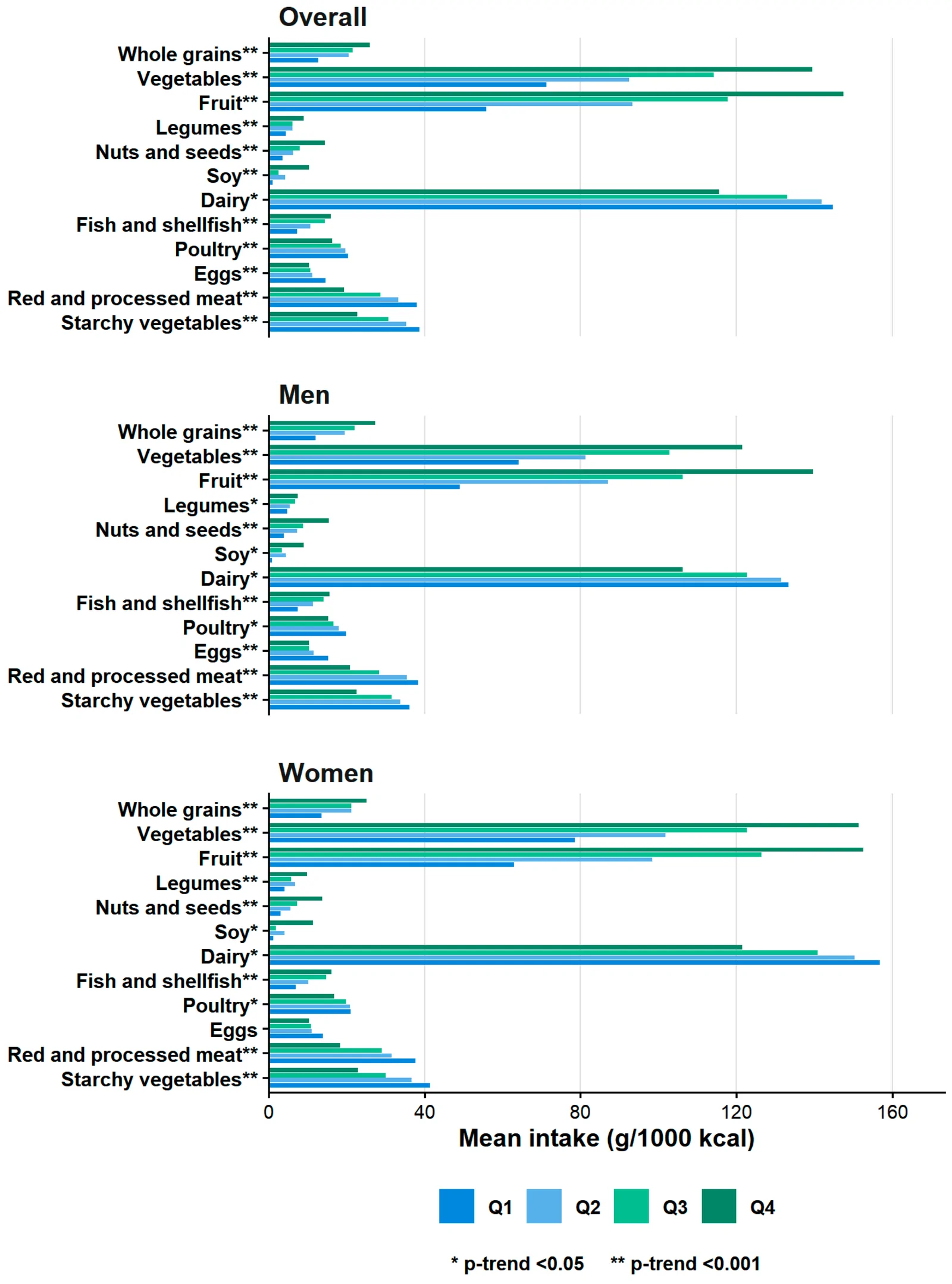
Survey-weighted mean standardized intake of food groups across quartiles of the Planetary Health Diet Index in the overall population and separately among men and women. Food-group intake is expressed as grams per 1000 kcal. Q1 and Q4 represent the lowest and the highest quartile. Bars represent survey-weighted means. Q1 = lowest PHDI adherence; Q4 = highest PHDI adherence. * *p*-trend < 0.05; ** *p*-trend < 0.001 from survey-weighted linear regression of each food group on PHDI quartile as a continuous variable. Detailed data are shown in Supplementary Tables S2–S4.

Across PHDI quartiles (Tables S1–S3 and Figure 3), intake of whole grains, vegetables, fruit, nuts and seeds, fish and shellfish, and added unsaturated fat increased progressively from Q1 to Q4, while red and processed meat, starchy vegetables, poultry, and added sugar decreased progressively; all of these trends were significant overall and in both sexes (all *p* for trend ≤ 0.006). Legume and soy intake was also significantly higher at higher PHDI levels overall and in both sexes (all *p* for trend ≤ 0.004), although quartile means were not strictly graded. Dairy intake likewise decreased across increasing PHDI quartiles, with significant trends in the overall population and in both sexes (*p* for trend = 0.002 overall, 0.021 in men, and 0.006 in women). Egg intake decreased significantly across quartiles overall and among men (both *p* for trend < 0.001), with a marginally significant trend among women (*p* for trend = 0.057).

### 3.3. Association Between PHDI and eGFR Decline

Results of the multivariable survey-weighted regression analyses examining the association between PHDI and continuous eGFR decline are presented in Table 3. In the overall sample, PHDI was not significantly associated with eGFR decline. Each 10-point-higher PHDI score was associated with a 0.218 mL/min/1.73 m^2^ per year less negative eGFR decline (95% CI −0.065 to 0.466; *p* = 0.133). Similarly, none of the individual PHDI quartile contrasts reached statistical significance, including Q4 versus Q1 (β = 0.608; 95% CI −0.368 to 1.584; *p* = 0.212), and the ordinal trend across quartiles was not significant (β per quartile = 0.225; 95% CI −0.071 to 0.520; *p* for trend = 0.131).

Sex-stratified analyses showed different patterns. Among men, the association was weak and not significant in both continuous analyses (per 10-point increase, β = 0.080; 95% CI −0.294 to 0.454; *p* = 0.665), with no significant association for Q4 versus Q1 (β = 0.237; 95% CI −1.003 to 1.478; *p* = 0.698) and no evidence of a linear trend across quartiles (β per quartile = 0.102; 95% CI −0.297 to 0.501; *p* for trend = 0.607). Among women, each 10-point-higher PHDI score was associated with a 0.355 mL/min/1.73 m^2^ per year less negative eGFR decline (95% CI 0.029 to 0.681; *p* = 0.034). Although none of the individual quartile contrasts reached statistical significance, the ordinal trend across increasing PHDI quartiles was slightly significant (*p* for trend = 0.036), indicating progressively slower eGFR decline with higher PHDI adherence among women.

Over the follow-up time, 404 participants (19.0%) experienced a relative decline in eGFR of at least 30%. The crude frequency of rapid decline was virtually identical in the two sexes, occurring in 180 men (18.9%) and 224 women (19.0%). The association between PHDI score and eGFR decline of at least 30% was investigated by using multivariable survey weighted logistic regression models (Table 4).

In the overall population, PHDI was not significantly associated with the risk of experiencing a ≥30% decline in eGFR. Each 10-point-higher PHDI score was associated with an OR of 0.933 (95% CI, 0.828, 1.051; *p* = 0.244), and neither the individual quartile comparisons nor the trend across quartiles reached statistical significance.

Among men, there was likewise no evidence of an association. The OR per 10-point-higher PHDI score was 1.080 (95% CI, 0.901–1.295; *p* = 0.394), and neither the individual quartile comparisons nor the trend across quartiles was statistically significant.

Among women, however, each 10-point-higher PHDI score was associated with a 20.1% lower odds of ≥30% eGFR decline (OR, 0.799; 95% CI, 0.687–0.930; *p* = 0.005). Women in Q4 had lower estimated odds than those in Q1 (OR, 0.599; 95% CI, 0.360–0.996; *p* = 0.048), whereas the Q2 and Q3 contrasts did not reach statistical significance. The trend across increasing PHDI quartiles was significant (OR per quartile increase, 0.837; 95% CI, 0.712–0.985; *p* for trend = 0.033). This sex-specific pattern is consistent with the significant multiplicative interaction between PHDI and sex for this outcome (*p* for interaction = 0.002).

Table 5 presents the additive interaction between PHDI and sex for the risk of a ≥30% decline in eGFR.

Among men, a 10-point-higher PHDI score was associated with a non-significant 1.33-percentage-point increase in absolute risk (95% CI, −0.79 to 3.44; *p* = 0.219). Among women, the same 10-point increase in PHDI was associated with a significant 3.03-percentage-point reduction in absolute risk (95% CI, −4.84 to −1.22; *p* = 0.001). The difference in absolute risk changes between women and men was −4.35 percentage points (95% CI, −6.90 to −1.80; *p* < 0.001), indicating significant interaction on the additive scale.

### 3.4. Sensitivity Analyses Using the Combined Creatinine–Cystatin C Equation

These analyses were restricted to the 1698 participants with both serum creatinine and cystatin C available at the 2016 examination. Higher adherence to the Planetary Health Diet Index (PHDI) was cross-sectionally associated with a modestly higher combined creatinine–cystatin C eGFR in the overall population, with each 10-point increment in PHDI corresponding to a 0.942 mL/min/1.73 m^2^ higher eGFR (95% CI −0.095 to 1.978; *p* = 0.074) (Table 6).

This association was modified by sex: among women, each 10-point PHDI increment was associated with a 1.34 mL/min/1.73 m^2^ higher eGFR (95% CI: 0.199, 2.482; *p* = 0.020), and women in the highest PHDI quartile (Q4) had an eGFR 3.48 mL/min/1.73 m^2^ higher than those in the lowest quartile (95% CI: −0.285, 7.244; *p* = 0.069), with a marginal linear trend across quartiles (*p* = 0.091). In contrast, no meaningful association was observed among men, either for the continuous PHDI score (β = 0.316, 95% CI: −1.131, 1.764; *p* = 0.658) or across quartiles (*p* for trend = 0.615).

When PHDI and sex were formally tested for a multiplicative interaction in a combined model, the interaction term did not reach statistical significance (F = 1.55, *p* = 0.222). On the additive scale, the sex-specific risk differences suggested a reduction in absolute risk among women (−2.58 percentage points per 10-point increase in PHD), with a statistically significant difference compared with men (−4.47 percentage points, 95% CI: −7.94, −0.99, *p* = 0.012), suggesting a plausible sex interaction on the additive scale (Table 6).

### 3.5. Sensitivity Analyses in Individuals with Baseline eGFR ≥ 60 mL/min/1.73 m^2^

To evaluate whether the observed associations could be influenced by reverse causation and baseline kidney dysfunction, primary analyses were repeated in the subgroup of 1453 individuals with baseline eGFR ≥ 60 mL/min/1.73 m^2^. For annualized eGFR change, the association among women remained significant and was numerically stronger than in the primary analysis, with a consistent graded pattern across quartiles (Table 7).

For the risk of a ≥30% eGFR decline, the protective association was significant in women and not in men (Table 8). The sex interaction remained significant on both the multiplicative and additive scales, with a substantially greater absolute risk reduction in women than in men.

## 4. Discussion

Where earlier studies of the Planetary Health Diet and the kidney asked whether adherence predicts incident CKD, the present analysis followed the rate at which kidney function declined over four years. Because the endpoint was a rate of change, the choice of filtration marker was especially important: serum creatinine follows skeletal muscle and can flatten the apparent decline as muscle is lost with age, so kidney function was estimated from cystatin C-based eGFR, which is less dependent on muscle. The same cohort allowed women and men to be examined within a single set of models, an approach seldom taken for a diet usually studied in mixed-sex or women-only samples, and the association proved to differ between the sexes, with effect modification on the multiplicative and additive scales. In this nationally representative cohort of US adults aged 50 years and older, adherence showed no significant association with decline in the sample as a whole; the associations, however, differed by sex. Among women, each 10-point-higher PHDI score was associated with slower eGFR decline in both continuous and categorical analyses, with graded trends across quartiles. Among men, neither outcome showed a significant benefit, and for a ≥30% decline the point estimate was in the opposite direction. The association between the Planetary Health Diet and kidney function decline in this cohort was therefore evident among women but not among men.

Evidence linking the PHD to kidney health has been based on both cross-sectional and longitudinal cohort studies but was generally based on the association between diet and the prevalence or incidence of CKD; indeed, cross-sectional analyses of NHANES showed that higher adherence to PHD was associated with improved kidney health and lower medication use in older adults with CKD [4,5].

In the UK Biobank, the highest adherence quintile was associated with a hazard ratio of 0.74 (95% CI 0.65–0.85) for incident CKD compared with the lowest [11]. Subsequent UK Biobank analyses reproduced this inverse association using alternative adherence scores. Yang and colleagues reported consistent risk reductions across four scoring methods in 179,508 participants, with adjusted hazard ratios between 0.91 and 0.94 [27], and Lv and colleagues found a comparable association that was further shaped by genetic susceptibility to CKD [13]. A joint analysis of the UK Biobank and the US National Health and Nutrition Examination Survey showed the same graded relationship in 119,820 and 19,991 participants, respectively, with a relative risk of 0.78 (95% CI 0.70–0.87) for the highest adherence category in NHANES and a hazard ratio of 0.71 (95% CI 0.64–0.79) for the top score quintile in the UK Biobank [28]. Finally, a recent multicentre study in the TCLSIH and UK Biobank cohorts similarly found that higher PHDI adherence was associated with a lower risk of incident CKD [12].

The overall non-significant result follows from this divergence: a benefit in women and a numerically opposite estimate in men offset one another in the pooled sample, masking an association that is present in one sex. Among women, the association was consistently observed across all analyses: the continuous eGFR decline, the graded trend across quartiles, the odds of a ≥30% decline, and the interaction tests on the multiplicative and additive scales. The consistency of the findings across multiple analytical approaches supports the robustness of the observed association, although confirmation in independent cohorts remains warranted. The individual quartile contrasts in women did not reach significance while the trends did, a pattern that fits a graded, dose-dependent association across the range of adherence rather than a threshold above which risk falls. Assessment of interaction on both the additive and multiplicative scales is relevant for clinical interpretation, because absolute risks bear directly on how many people are affected: a 10-point-higher score corresponded to a 2.54-percentage-point-lower absolute risk of a ≥30% decline in women and to a non-significant increase in men, a between-sex difference of −4.02 percentage points (95% CI −6.54 to −1.50). This sex-specific pattern is directionally consistent with a recent multicentre study in the TCLSIH and UK Biobank cohorts [12], which reported a stronger PHDI-related protection against incident CKD in women than in men, with evidence of a sex interaction. That study, however, examined incident CKD as a diagnostic threshold, which by definition can only capture change in participants who cross that threshold and cannot distinguish a slowing of decline from no decline at all. By modeling continuous and categorical eGFR change, the present study captures the sex-specific association across the full range of renal trajectories, including among participants who do not develop diagnosable CKD. More importantly, the magnitude of the slower eGFR decline observed in women of our study (0.35 mL/min/1.73 m^2^/year per 10-point PHDI increase) is statistically significant and represents a relevant proportion of the typical age-related eGFR decline in older adults (approximately 0.5–1 mL/min/1.73 m^2^/year); if sustained over a longer period than our 4-year follow-up, a difference of this magnitude can translate into a delayed progression toward clinically relevant eGFR thresholds; furthermore, this continuous effect was accompanied by a comparable association with the ≥ 30% eGFR decline threshold, a change generally regarded as clinically significant, further supporting the clinical relevance of our study findings.

Earlier work on the rate of decline was conducted before the PHD was proposed and relied on creatinine. In the Nurses’ Health Study a Western pattern was associated with rapid eGFR loss of at least 3 mL/min/1.73 m^2^ per year (odds ratio 1.77; 1.03 to 3.03) and a DASH pattern with lower risk (odds ratio 0.55; 0.38 to 0.80) in a sample confined to older white women [16]. Where decline in older adults was instead assessed with cystatin C, the exposure was protein intake, which showed no association with rapid decline [29].

Longitudinal evidence testing effect modification by sex in the diet–kidney health association has instead been scarce, though it aligns with our results: in a study of two cohorts, greater diet adherence was associated with lower incident CKD among women, with no clear association in men [12]. The present analysis extends this using a different endpoint, represented by the rate of decline rather than the crossing of a diagnostic threshold, and a filtration marker, cystatin C, that is less dependent on muscle mass and protein intake. Among women, each 10-point-higher score was associated with a lower eGFR decline during the follow-up and with 20% lower odds of a ≥30% decline (OR 0.799; 95% CI 0.687 to 0.930), whereas the corresponding estimates in men were non-significant (β 0.080; 95% CI −0.294 to 0.454; OR 1.080; 95% CI 0.901 to 1.295). This pattern is consistent with the flatter age-related decline of filtration described in women [14]. The difference between the sexes was observed in the present study and supported by significance of interaction terms, but awaits confirmation in further studies. The muscle and adiposity contributions to the two markers also differ between women and men, with dietary protein associated with the creatinine–cystatin discordance in men [19].

Greater adherence to the PHD may be associated with a lower cystatin C-based eGFR decline, potentially reflecting preservation of glomerular filtration or lower adiposity and inflammation that raise cystatin C independently of filtration. Even when GFR is measured directly the candidate non-GFR determinants account for only a minority of the variance in the creatinine–cystatin difference [30], so the markers resist clean separation. In adults with diabetes, cystatin C carried more of the association between a plant-based diet and CKD than creatinine, accounting for 47% and 89% of the association for healthful and less healthful plant-based diets, against 39% and 44% for creatinine [31], consistent with part of the diet signal residing on the cystatin side. Higher adherence has been linked to lower adiposity and inflammation [11,27] and to routes nearer filtration, including a higher dietary alkali load and a fibre-associated shift in the gut microbiome [32]. Whether the slower decline observed here reflects filtration, these non-GFR pathways, or both cannot be resolved from a single marker.

In sensitivity analyses at the 2016 examination, kidney function estimated with the combined creatinine–cystatin C equation showed the same directional pattern, with higher adherence associated with better kidney function chiefly among women. When both filtration markers are available, this combined estimate is the most accurate single measure of glomerular filtration against measured GFR [26,33] and is the form recommended for a single assessment [8]. The women-specific association was thus reproduced with a marker set that includes creatinine, which argues against its being an artefact of the non-GFR determinants of cystatin C alone. Formal interaction by sex did not reach significance in this cross-sectional analysis, so the sex difference was clearer when decline was followed longitudinally with cystatin C than when filtration was assessed once with the combined marker. However, beyond statistical significance, the underlying pattern was similar to the primary analysis: the association trended and more consistently positive in women than men, mirroring the direction observed in longitudinal models. Notably, the additive-scale sex interaction persisted and remained statistically significant in this sensitivity analysis as well, consistent with our primary longitudinal findings. Taken together, these findings suggest that the observed association is robust across different approaches to estimating kidney function, although the longitudinal cystatin C analyses remain the primary evidence because they evaluate change over time rather than a single cross-sectional assessment. In line with guidance for settings where creatinine-based estimates may be unreliable, cystatin C-based or combined estimates warrant consideration when kidney disease progression is assessed in older adults.

Several biological mechanisms have been proposed in the literature to explain sex differences in kidney function decline, though none were directly assessed in this cohort, and no study to our knowledge has directly tested their role as potential contributors to sex-specific associations between diet and kidney function. Estrogens have shown to exert protective hemodynamic effects on the glomeruli, restrain the renin–angiotensin system, temper renal inflammation and fibrosis, and support antioxidant capacity [34,35], thus potentially amplifying the anti-inflammatory effects of PHD; estrogen fall after menopause coincides with faster loss of kidney function, so the ageing female kidney may retain more scope to respond to a favourable dietary pattern [34,35]. Another potential contributor may be represented by the beneficial effects of plant-based diets on gut health, which in turn regulate kidney function: a plant-forward pattern shifts the microbiome towards fibre-fermenting taxa that generate short-chain fatty acids [32]; both the composition of the microbiome and its two-way exchange with estrogen differ between women and men [36], so the same change in diet may produce different downstream effects in each sex. The higher polyphenol and antioxidant content of a Planetary Health Diet may in turn act alongside the antioxidant effects of estrogen [34].

However, whether these pathways may contribute to the sex-specific association observed in this study has not been tested and remains speculative. We did not measure hormonal status, oxidative stress biomarkers, or gut microbiome composition in this cohort, and all of these mechanisms should be regarded as hypotheses for targeted future studies.

This analysis draws on a nationally representative cohort of US adults aged 50 years and older, with diet captured by a validated questionnaire and kidney function by repeated cystatin C-based eGFR, which allowed an individual rate of change to be estimated and compared between women and men in one model. Because all models were survey-weighted, the estimates account for the study’s complex sampling design and apply to the community-dwelling US population aged 50 years and older.

This study has several limitations. Diet was measured once, in 2013, using a food frequency questionnaire covering the preceding 12 months; this single assessment could not capture within-person dietary change over the subsequent follow-up, thus potentially leading to misclassification; additionally, dietary assessment followed the 2012 baseline cystatin C measurement by approximately one year, so we cannot fully exclude reverse causation, that is, participants with early, subclinical decline in kidney function modifying their diet before completing the questionnaire; however, when the analysis was restricted to participants with baseline eGFR ≥ 60 mL/min/1.73 m^2^, the protective association among women remained significant and was numerically stronger, with preservation of sex interactions, thereby supporting the robustness of the observed association independent of baseline kidney function. The eGFR decline derives mainly from repeated dried blood spot cystatin C, which carries the assay variability of dried blood spots and documented changes over time in assay protocols and platforms across laboratories [21], so each individual estimate was uncertain. Additionally, the 2016 cystatin C measurement combined DBS and VBS sources for participants missing the former; agreement between the two was good but not complete (r = 0.87), introducing modest additional measurement heterogeneity beyond dried blood spot assay variability alone. Cystatin C reflects filtration together with adiposity and inflammation, and part of the association may run through these non-GFR determinants. Furthermore, kidney function decline was estimated from only two cystatin C measurements four years apart, which is more susceptible to measurement error and regression to the mean than models based on three or more repeated measures. The cohort comprised adults aged 50 years and older, and the findings need not extend to younger adults, non-US populations, or patients with advanced CKD. As an observational study, residual confounding remains possible. To this regard, we were unable to account for use of specific medication classes such as angiotensin converting enzyme (ACE) inhibitors, Sodium–Glucose Cotransporter-2 (SGLT2) inhibitors, or statins, or for proteinuria, as these are not captured at the relevant waves in the HRS/HCNS data source; we did, however, incorporate broader indicators of cardiac, antidiabetic, and antihypertensive medication use, along with healthcare utilization and socioeconomic status. Sensitivity analysis with the combined creatinine–cystatin C equation was cross-sectional and performed in a smaller subset of participants, limiting direct comparison with the longitudinal cystatin C analyses. Finally, some findings and interactions with marginal statistical significance should be interpreted as exploratory, pending independent replication in larger samples. Beyond the specific findings of this study, our experience highlights broader considerations for the design of future longitudinal research on diet and kidney health. Repeated dietary assessment aligned with biomarker collection would help capture within-person dietary change over time and strengthen causal inference. Additional kidney function measurements beyond two time points would improve the precision of estimated decline rates. Richer data on medication use and proteinuria would support more comprehensive confounder adjustment in future studies of this kind.

## 5. Conclusions

In US adults aged 50 years and older, the association between adherence to the Planetary Health Diet and kidney function decline differed between women and men. Among women, greater adherence was associated with slower cystatin C-based eGFR decline and lower odds of a ≥30% decline across continuous models and graded quartile trends, whereas the corresponding estimates in men were weaker and, for the binary outcome, directionally opposite, although neither reached statistical significance. The significant interaction by sex suggests that sex may act as a biological modifier of the association between diet quality and kidney ageing rather than merely a confounder requiring statistical adjustment. This supports considering sex explicitly in nutritional epidemiology and in nephroprotective dietary strategies. As an observational analysis with a single dietary assessment, and because a non-significant result in men does not establish the absence of an effect, these findings require confirmation in prospective studies with repeated dietary assessment and, where feasible, directly measured GFR. Sensitivity analyses using the combined creatinine–cystatin C equation yielded directionally consistent findings, supporting the robustness of the women-specific association.

## Figures and Tables

**Table 1:** Baseline characteristics of the analytic sample overall and by sex.

	Overall (n = 2126)	Men (n = 950)	Women (n = 1176)	*p*-Value
Age, mean (SD)	64.3 (9.2)	64.0 (9.2)	64.6 (9.2)	0.162
Race, n (%)				0.826
Non-Hispanic White	1683 (79.2)	758 (79.8)	925 (78.7)	
Non-Hispanic Black	195 (9.2)	81 (8.5)	114 (9.7)	
Hispanic	177 (8.3)	79 (8.4)	98 (8.3)	
Other	71 (3.3)	32 (3.3)	39 (3.3)	
Poverty–income ratio, median (IQR)	3.9 (2.1–6.5)	4.2 (2.4–7.0)	3.7 (2.0–6.1)	<0.001
Number of annual medical visits, median (IQR)	5 (2–10)	5 (2–9)	6 (3–10)	<0.001
Education years, mean (SD)	13.3 (2.9)	13.4 (2.9)	13.2 (2.8)	0.047
BMI, mean (SD)	28.8 (5.9)	28.6 (5.0)	28.9 (6.5)	0.287
Current smoker, n (%)	288 (13.6)	142 (15.0)	146 (12.5)	0.107
Days per week of alcohol consumption, mean (SD)	1.35 (2.1)	1.74 (2.3)	1.04 (1.9)	<0.001
Moderate-vigorous physical activity, n (%)	1621 (76.3)	780 (82.2)	841 (71.5)	<0.001
Hypertension, n (%)	1107 (52.1)	502 (52.9)	605 (51.4)	0.631
Type 2 diabetes, n (%)	393 (18.5)	193 (20.3)	200 (17.0)	0.092
Cardiovascular disease, n (%)	411 (19.3)	206 (21.7)	205 (17.4)	0.011
Stroke, n (%)	124 (5.9)	69 (7.3)	55 (4.7)	0.045
Cancer, n (%)	299 (14.1)	134 (14.1)	165 (14.0)	0.959
Chronic lung disease, n (%)	177 (8.3)	77 (8.1)	100 (8.5)	0.747
CES-D, mean (SD)	1.3 (2.0)	1.2 (1.9)	1.4 (2.0)	0.019
Number of drugs, mean (SD)	0.8 (0.4)	0.8 (0.4)	0.8 (0.4)	0.437
Medications for heart disease, n (%)	321 (15.1)	177 (18.6)	144 (12.3)	<0.001
Antihypertensive medications, n (%)	1018 (47.9)	465 (49.0)	553 (47.0)	0.465
Antidiabetic medications, n (%)	318 (15.0)	158 (16.7)	160 (13.6)	0.104
eGFR, mL/min/1.73 m^2^, mean (SD)	73.9 (26.0)	76.3 (25.9)	72.0 (25.9)	0.004
eGFR stages, mL/min/1.73 m^2^, n (%)				0.005
≥60	1453 (68.4)	684 (72.1)	769 (65.4)	
45–59.9	391 (18.4)	169 (17.7)	222 (18.9)	
30–44.9	211 (9.9)	73 (7.7)	138 (11.7)	
<30	71 (3.3)	24 (2.5)	47 (4.0)	
PHDI score, mean (SD)	77.2 (12.3)	75.9 (12.3)	78.3 (12.1)	<0.001

Notes: BMI, body mass index; CES-D, Center for Epidemiologic Studies Depression scale; eGFR, estimated glomerular filtration rate; PHDI, Planetary Health Diet Index.

**Table 2:** Survey-weighted mean (SD) daily food group intake standardized per 1000 kcal (g/1000 kcal), overall and by sex.

Food Group	Overall, Mean (SD)	Males, Mean (SD)	Females, Mean (SD)	*p*-Value
Whole grains	20.2 (13.8)	19.7 (14.0)	20.6 (13.6)	0.152
Vegetables	104.4 (62.7)	90.4 (54.3)	115.6 (66.6)	<0.001
Fruit	103.6 (76.9)	92.4 (72.4)	112.6 (79.2)	<0.001
Legumes	6.4 (8.9)	6.0 (8.5)	6.7 (9.2)	0.117
Nuts and seeds	8.0 (8.4)	8.4 (8.5)	7.7 (8.4)	0.157
Soy	4.6 (24.2)	4.1 (23.4)	4.9 (24.8)	0.542
Dairy	133.8 (122.6)	124.4 (103.8)	141.4 (135.4)	0.002
Fish and shellfish	12.1 (10.3)	11.8 (9.6)	12.3 (10.8)	0.372
Poultry	18.7 (15.4)	17.6 (14.7)	19.6 (16.0)	0.004
Eggs	11.7 (13.4)	12.0 (11.7)	11.4 (14.7)	0.441
Red and processed meat	29.8 (17.6)	31.3 (17.6)	28.6 (17.5)	<0.001
Starchy vegetables	31.9 (22.9)	31.4 (22.0)	32.2 (23.6)	0.385
Added unsaturated fat	10.7 (5.0)	10.6 (4.7)	10.8 (5.2)	0.519
Added saturated and trans fat	16.9 (5.2)	16.8 (5.2)	17.0 (5.3)	0.363
Added sugar	28.5 (17.8)	28.8 (17.3)	28.3 (18.3)	0.636

**Table 3:** Survey-weighted linear regression models exploring the association between the Planetary Health Diet Index (PHDI) and the cystatin C-based eGFR decline (in mL/min/1.73 m^2^ per year) in the whole study population, men and women. Positive coefficients indicate a slower eGFR decline.

PHDI Exposure	Overall, Estimate (95% CI)	*p*-Value	Men, Estimate (95% CI)	*p*-Value	Women, Estimate (95% CI)	*p*-Value
Per 10-point increase	0.218 (−0.065, 0.466)	0.133	0.080 (−0.294, 0.454)	0.665	0.355 (0.029, 0.681)	0.034
Q2 vs. Q1	0.041 (−0.830, 0.912)	0.924	0.349 (−0.926, 1.624)	0.580	−0.139 (−1.122, 0.845)	0.775
Q3 vs. Q1	0.465 (−0.324, 1.254)	0.238	0.547 (−0.597, 1.691)	0.336	0.529 (−0.388, 1.446)	0.248
Q4 vs. Q1	0.608 (−0.368, 1.584)	0.212	0.237 (−1.003, 1.478)	0.698	0.943 (−0.153, 2.039)	0.089
*p* for trend	0.225 (−0.071, 0.520)	0.131	0.102 (−0.297, 0.501)	0.607	0.359 (0.024, 0.693)	0.036

Notes: PHDI = Planetary Health Diet Index; Q = quartile.

**Table 4:** Survey-weighted logistic regression models exploring the association between the Planetary Health Diet Index (PHDI) and the odds of a relative eGFR decline of at least 30% between 2012 and 2016 in the whole study population, men and women.

PHDI Exposure	Overall, OR (95% CI)	*p*-Value	Men, OR (95% CI)	*p*-Value	Women, OR (95% CI)	*p*-Value
Per 10-point increase	0.933 (0.828–1.051)	0.244	1.080 (0.901–1.295)	0.394	0.799 (0.687–0.930)	0.005
Q2 vs. Q1	1.091 (0.746–1.593)	0.644	1.318 (0.694–2.501)	0.385	0.975 (0.624–1.525)	0.909
Q3 vs. Q1	1.032 (0.690–1.516)	0.907	1.388 (0.753–2.558)	0.281	0.774 (0.491–1.219)	0.259
Q4 vs. Q1	0.800 (0.510–1.256)	0.320	1.034 (0.524–2.041)	0.919	0.599 (0.360–0.996)	0.048
*p* for trend	0.935 (0.815–1.061)	0.271	1.021 (0.838–1.245)	0.829	0.837 (0.712–0.985)	0.033
PHDI*Sex	F = 11.14	0.002				

**Table 5:** Sex-specific absolute risk differences and additive interaction for the association between PHDI and ≥30% eGFR decline. Values are expressed in percentage points.

Group	Absolute Risk Difference per 10-Point-Higher PHDI (95% CI)	*p*-Value
Men	1.33 (−0.79 to 3.44)	0.219
Women	−3.03 (−4.84 to −1.22) pp	0.001
Women vs. men difference	−4.35 (−6.90 to −1.80) pp	<0.001

Notes: pp = percentage points.

**Table 6:** Survey-weighted linear regression exploring the association between PHDI and follow-up eGFRcys-cre among 1698 individuals with available data on follow-up creatinine.

PHDI Exposure	Overall β (95% CI)	*p*	Men β (95% CI)	*p*	Women β (95% CI)	*p*
Per 10-point increment	0.942 (−0.095, 1.978)	0.074	0.316 (−1.131, 1.764)	0.658	1.340 (0.199, 2.482)	0.023
Q2 vs. Q1	1.584 (−1.607, 4.775)	0.319	2.198 (−2.870, 7.267)	0.380	0.850 (−2.680, 4.381)	0.626
Q3 vs. Q1	1.077 (−2.269, 4.423)	0.516	1.713 (−2.332, 5.758)	0.391	0.692 (−3.401, 4.786)	0.732
Q4 vs. Q1	2.728 (−0.634, 6.089)	0.108	1.217 (−3.278, 5.713)	0.582	3.480 (−0.285, 7.244)	0.069
Linear trend across quartiles	0.771 (−0.323, 1.865)	0.161	0.356 (−1.079, 1.791)	0.615	1.059 (−0.177, 2.295)	0.091
PHDI × Sex interaction	F = 1.55 (*df* = 1.31)	0.222				
Risk difference in pp for 10-point PHDI change (95% CI)	Women-men; −4.471 (−7.943, −0.992)	0.012	1.881 (−0.970, 4.73)	0.195	−2.582 (−5.181, 0.022)	0.050

Notes: PHDI = planetary health diet index; pp = percentage points; Q = quartile.

**Table 7:** Association between PHDI and annualized eGFR change among 1453 individuals with baseline eGFR_2012_ ≥ 60 mL/min/1.73 m^2^.

PHDI Exposure	Overall β (95% CI)	*p*	Men β (95% CI)	*p*	Women β (95% CI)	*p*
Per 10-point increment	0.262 (−0.019, 0.542)	0.067	0.065 (−0.314, 0.445)	0.726	0.556 (0.148, 0.963)	0.009
Q2 vs. Q1	0.496 (−0.582, 1.574)	0.354	0.290 (−1.254, 1.835)	0.701	0.821 (−0.509, 2.151)	0.217
Q3 vs. Q1	0.767 (−0.214, 1.749)	0.121	0.304 (−1.222, 1.830)	0.684	1.384 (0.196, 2.572)	0.024
Q4 vs. Q1	0.883 (−0.166, 1.931)	0.096	0.295 (−1.109, 1.699)	0.669	1.711 (0.341, 3.081)	0.016
Linear trend across quartiles	0.287 (−0.031, 0.604)	0.076	0.094 (−0.347, 0.536)	0.664	0.550 (0.136, 0.964)	0.011

Notes: PHDI = Planetary Health Diet Index; Q = quartile.

**Table 8:** Association between PHDI and longitudinal ≥30% eGFR decline among 1453 individuals with baseline eGFR_2012_ ≥ 60 mL/min/1.73 m^2^.

	Overall OR (95% CI)	*p*	Men OR (95% CI)	*p*	Women OR (95% CI)	*p*
Per 10-point increment	0.933 (0.812, 1.071)	0.312	1.134 (0.908, 1.416)	0.256	0.737 (0.604, 0.900)	0.004
Q2 vs. Q1	1.034 (0.636, 1.680)	0.889	1.508 (0.633, 3.594)	0.339	0.682 (0.370, 1.257)	0.211
Q3 vs. Q1	1.035 (0.648, 1.654)	0.880	1.551 (0.635, 3.787)	0.321	0.637 (0.347, 1.171)	0.140
Q4 vs. Q1	0.781 (0.467, 1.306)	0.334	1.113 (0.489, 2.535)	0.790	0.466 (0.248, 0.874)	0.019
Linear trend across quartiles	0.926 (0.795, 1.080)	0.317	1.038 (0.818, 1.316)	0.752	0.791 (0.644, 0.972)	0.027
PHDI × sex interaction	F = 12.44 (*df* = 1.29), *p* = 0.001					
Risk difference in pp for 10-point PHDI change (95% CI)	Women-men: −5.99 (−9.21, −2.75)	<0.001	1.74 (−0.71, 4.20)	0.164	−4.25 (−6.63, −1.87)	<0.001

Notes: PHDI = Planetary Health Diet Index; Q = quartile; pp = percentage points.

## Data Availability

The data used in this study are available from the Health and Retirement Study (HRS), sponsored by the National Institute on Aging and conducted by the University of Michigan. HRS data are available to qualified researchers upon registration and execution of a Data Use Agreement via the HRS data portal (https://hrs.isr.umich.edu).

## Supplementary Materials

The following supporting information can be downloaded at: https://www.mdpi.com/article/10.3390/nu18172850/s1, Table S1. Components of the Planetary Health Diet Index (PHDI), EAT–Lancet reference diet targets, scoring criteria, and the specific HCNS Food Frequency Questionnaire (FFQ) items and gram-per-serving conversion factors used to construct each component; Table S2. Survey-weighted standardized food-group intake and added fat and sugar consumption across PHDI quartiles in the overall population; Table S3. Survey-weighted standardized food-group intake and added fat and sugar consumption across PHDI quartiles among men; Table S4. Survey-weighted standardized food-group intake and added fat and sugar consumption across PHDI quartiles among women.

## Abbreviations

The following abbreviations are used in this manuscript:

ACE	Angiotensin converting enzyme
BMI	Body mass index
CES-D	Center for Epidemiologic Studies Depression scale
CI	Confidence interval
CKD	Chronic kidney disease
CKD-EPI	Chronic Kidney Disease Epidemiology Collaboration
DASH	Dietary Approaches to Stop Hypertension
DBS	Dried blood spot
eGFR	Estimated glomerular filtration rate
FFQ	Food frequency questionnaire
GFR	Glomerular filtration rate
HCNS	Health Care and Nutrition Study
HRS	Health and Retirement Study
NHANES	National Health and Nutrition Examination Survey
OR	Odds ratio
PHD	Planetary Health Diet
PHDI	Planetary Health Diet Index
Q	Quartile
SD	Standard deviation
SMD	Standardized Mean Difference
SGLT2	Sodium-Glucose Cotransporter-2
VBS	Venous blood sample
