# Supplementary material for: Sex Modifies the Association Between Planetary Health Diet Adherence and Kidney Function Decline in US Adults Aged 50 Years and Older
Source: Nutrients. 2026 Sep 1;18(17):2850. doi: 10.3390/nu18172850 (PMC13567324; doi:10.3390/nu18172850)
Supplement: Supplementary file 1 [file nutrients-18-02850-s001.zip › nutrients-4500932-supplementary.pdf]

Table S1. Components of the Planetary Health Diet Index (PHDI), EAT–Lancet reference diet targets, scoring criteria, and the specific HCNS Food Frequency Questionnaire (FFQ) items and gram-per-serving conversion factors used to construct each component.

PHDI component / scoring criteria	Example FFQ food item	Grams per 1 serving	HCNS FFQ item code
**Whole grains (dry weight)**<br>*EAT–Lancet reference: 232 g/d (0–60% of energy)*<br>*Min score (0): 0 g/d ∣ Max score: ≥75 g/d (women); ≥90 g/d (men)*			
	Whole grain	—	WGRNSUM (pre-aggregated HCNS variable, not summed from individual FFQ items)
**Starchy vegetables (e.g., potatoes, cassava)**<br>*EAT–Lancet reference: 50 g/d (0–100)*<br>*Min score (0): ≥200 g/d ∣ Max score: ≤50 g/d*			
	French fried potatoes (6 oz)	170	C7P_FF

PHDI component / scoring criteria	Example FFQ food item	Grams per 1 serving	HCNS FFQ item code
	Potatoes, baked, boiled or mashed (1 cup)	177.41	C7Q_FF
	Yams or sweet potatoes (1/2 cup)	110.32	C5S_FF
	Potato or corn chips (1 oz)	28	C7R_FF
**Vegetables (excluding starchy vegetables)**<br>*EAT–Lancet reference: 300 g/d (200–600)*<br>*Min score (0): 0 g/d | Max score: ≥300 g/d*			
	Tomatoes (2 slices)	40	C5A_FF
	Tomato juice (small glass)	182	C5B_FF
	Tomato sauce (1/2 cup)	128	C5C_FF
	Green/snap/string beans (1/2 cup)	68	C5F_FF
	Peas or lima beans (1/2 cup fresh)	81.25	C5N_FF
	Broccoli (1/2 cup)	78	C5G_FF

PHDI component / scoring criteria	Example FFQ food item	Grams per 1 serving	HCNS FFQ item code
	Cauliflower (1/2 cup)	62	C5I_FF
	Cabbage or coleslaw (1/2 cup)	70.23	C5H_FF
	Brussels sprouts (1/2 cup)	78	C5J_FF
	Carrots, raw (1/2 carrot)	36	C5K_FF
	Carrots, cooked (1/2 cup)	78	C5L_FF
	Corn (1 ear or 1/2 cup)	82	C5M_FF
	Mixed vegetables (1/2 cup)	91	C5O_FF
	Winter squash, yellow/orange (1/2 cup)	102	C5Q_FF
	Eggplant, zucchini or other summer squash (1/2 cup)	90	C5R_FF
	Kale, mustard green or chard (1/2 cup)	68	C5V_FF
	Spinach, raw (1 cup)	30	C5U_FF
	Spinach, cooked (1/2 cup)	90	C5T_FF
	Iceberg or head lettuce (serving)	89	C5X_FF

PHDI component / scoring criteria	Example FFQ food item	Grams per 1 serving	HCNS FFQ item code
	Romaine or leaf lettuce (serving)	85	C5W_FF
	Celery (2–3 sticks)	20	C5Y_FF
	Green, yellow or red pepper (3 slices)	30	C5Z_FF
	Cooked onions (1/2 cup)	110	C5AB_FF
	Raw onions (1 slice)	14	C5AA_FF
**Whole fruit (excluding fruit juice)** *EAT–Lancet reference: 200 g/d (100–300)* *Min score (0): 0 g/d* | *Max score: ≥200 g/d*			
	Raisins (1 oz/small pack) or grapes (1/2 cup)	57.76	C4A_FF
	Prunes (6 dried or 1/2 cup canned)	59.28	C4B_FF
	Bananas (1)	118	C4D_FF
	Cantaloupe (1/4)	138	C4E_FF

**PHDI component / scoring criteria**	**Example FFQ food item**	**Grams per 1 serving**	**HCNS FFQ item code**
	Avocado (1/2 fruit)	100	C4F_FF
	Fresh apples/pears (1)	180.96	C4H_FF
	Applesauce (1/2 cup)	217	C4G_FF
	Oranges (1)	131	C4J_FF
	Grapefruit (1/2 cup)	128	C4M_FF
	Strawberries (1/2 cup, fresh/frozen/canned)	76	C4P_FF
	Other berries (1/2 cup, fresh/frozen/canned)	74	C4Q_FF
	Peaches or plums (1 fresh or 1/2 cup canned)	116.5	C4R_FF
	Apricots (1/2 cup canned)	39.07	C4S_FF
**Dairy foods (e.g., milk, cheese)**<br>*EAT–Lancet reference: 250 g/d (0–500)*<br>*Min score (0): ≥1000 g/d | Max score: ≤250 g/d*			

**PHDI component / scoring criteria**	**Example FFQ food item**	**Grams per 1 serving**	**HCNS FFQ item code**
	Skim milk (8 oz glass)	245	C3A_FF
	1% or 2% milk (8 oz glass)	244	C3B_FF
	Whole milk (8 oz glass)	244	C3C_FF
	Cream (1 tbsp)	8	C3E_FF
	Frozen yogurt / low-fat ice cream (1 cup)	143.42	C3G_FF
	Regular ice cream (1 cup)	132	C3H_FF
	Flavored yogurt (1 cup)	170	C3I_FF
	Low-carb yogurt (1 cup)	170	C3J_FF
	Cottage/ricotta cheese (1/2 cup)	113	C3L_FF
	Cream cheese (1 oz)	28	C3M_FF
	Other cheese (1 oz)	28	C3N_FF
**Red and processed meat (e.g., beef, lamb, pork)**<br><br>*EAT–Lancet reference: 14 g/d (0–28)*			

PHDI component / scoring criteria	Example FFQ food item	Grams per 1 serving	HCNS FFQ item code
Min score (0): ≥100 g/d | Max score: ≤14 g/d			
	Beef or pork hot dogs (1)	46.05	C6H_FF
	Bacon (2 slices)	16	C6D_FF
	Other processed meat (1 piece)	54	C6K_FF
	Processed meats (1 slice/piece)	56	C6J_FF
	Hamburger (1 patty)	85	C6M_FF
	Beef/pork/lamb sandwich or mixed dish	84.54	C6N_FF
	Pork as main dish (4–6 oz)	140.78	C6O_FF
	Beef or lamb as main dish (4–6 oz)	140	C6P_FF
	Liver, beef or pork (4 oz)	114	C6Q_FF
**Chicken and other poultry (duck, goose, ostrich...)** *EAT–Lancet reference: 29 g/d (0–58)*			

PHDI component / scoring criteria	Example FFQ food item	Grams per 1 serving	HCNS FFQ item code
Min score (0): ≥100 g/d ∣ Max score: ≤29 g/d			
	Chicken or turkey, frozen dinner (1)	85	C6E_FF
	Chicken or turkey, with skin (3 oz)	85	C6F_FF
	Chicken or turkey, without skin (3 oz)	85	C6G_FF
	Chicken or turkey hot dogs (1)	50.72	C6I_FF
	Liver, chicken or turkey (1 oz)	28	C6R_FF
**Eggs (with shell)**<br>*EAT–Lancet reference: 13 g/d (0–25)*<br>*Min score (0): ≥120 g/d ∣ Max score: ≤13 g/d*			
	Regular eggs, including yolk (1)	50	C6C_FF
	Fortified eggs, including yolk (1)	50	C6B_FF
**Fish and shellfish**<br>*EAT–Lancet reference: 28 g/d (0–100)*			

**PHDI component / scoring criteria**	**Example FFQ food item**	**Grams per 1 serving**	**HCNS FFQ item code**
Min score (0): 0 g/d ǀ Max score: ≥28 g/d			
	Canned tuna (3–4 oz)	99	C6S_FF
	Fish sticks (1 serving)	91	C6T_FF
	Shrimp, lobster, scallops as main dish	85	C6U_FF
	Dark-meat fish (3–5 oz)	113	C6V_FF
	Other fish (3–5 oz)	113	C6W_FF
**Nuts (peanuts, walnut, almond, hazelnut, pecan, cashew, pistachio)**<br>*EAT–Lancet reference: 50 g/d (0–75)*<br>*Min score (0): 0 g/d ǀ Max score: ≥50 g/d*			
	Peanut butter (1 tbsp)	16	C9F_FF
	Peanuts (1 oz)	28	C9V_FF
	Walnuts (1 oz)	28	C9W_FF

PHDI component / scoring criteria	Example FFQ food item	Grams per 1 serving	HCNS FFQ item code
	Other nuts (1 oz)	28	C9X_FF
**Added fat — unsaturated oils (e.g., olive, soybean, rapeseed, sunflower, peanut; excludes trans fat)**<br>*EAT–Lancet reference: 40 g/d (20–80)*<br>*Min score (0): ≤3.5% of energy intake ∣ Max score: ≥21% of energy intake (~60 g/d)*			
	Total vegetable fat minus total trans fat (mainly from oils), cohort-derived	—	ADDFAT_LIQ_SUM
**Added fat — saturated and trans fat (e.g., palm oil, coconut oil, butter, margarine, lard, tallow)**<br>*EAT–Lancet reference: 11.8 g/d (0–11.8)*<br>*Min score (0): ≥10% of energy intake ∣ Max score: 0% of energy intake*			

PHDI component / scoring criteria	Example FFQ food item	Grams per 1 serving	HCNS FFQ item code
	Dairy fat + total trans fat (+ palm/coconut oil if available), cohort-derived	—	ADDFAT_SOL_SUM
**Added sugar (all sweeteners and fruit juice)**<br>*EAT–Lancet reference: 31 g/d (0–31)*<br>*Min score (0): ≥25% of energy* | *Max score: ≤5% of energy*			
	Added sugar, g/day	—	ADDSUG_SUM (component 1)
	Sugar from juice, g/day	—	ADDSUG_SUM (component 2)
**Non-soy legumes (e.g., dry beans, lentils, peas) — weighted 0.5×**<br>*EAT–Lancet reference: 50 g/d (0–100)*<br>*Min score (0): 0 g/d* | *Max score: 100 g/d*			
	Beans or lentils (1/2 cup)	115.47	C5P_FF

PHDI component / scoring criteria	Example FFQ food item	Grams per 1 serving	HCNS FFQ item code
**Soybean / soy foods (dry weight) — weighted 0.5×**<br>*EAT–Lancet reference: 25 g/d (0–50)*<br>*Min score (0): 0 g/d | Max score: 50 g/d*			
	Tofu / soy protein (3–4 oz)	94.48	C5E_FF
	Soy milk (8 oz glass)	240	C3D_FF

Notes: Whole grains and the two added-fat components (unsaturated; saturated/trans) were derived from pre-aggregated HCNS nutrient-total variables (WGRNSUM, ADDFAT_LIQ_SUM, ADDFAT_SOL_SUM) rather than summed from individual FFQ items. Added sugar (ADDSUG_SUM) combines added-sugar and fruit-juice-sugar contributions. Non-soy legumes and soy foods are each weighted at 0.5 in the total PHDI score (maximum 5 points each), consistent with the implementation of the original PHDI algorithm in the dietaryindex R package (Zhan et al., Am J Clin Nutr 2024;120:1165-1174); all other components are weighted at 1.0 (maximum 10 points each), for a total possible PHDI score of 140. Gram-per-serving conversion factors reflect the portion size specified in the HCNS Food Frequency Questionnaire wording (e.g., "1/2 cup," "3 oz") and were derived from USDA Reference Database for Dietary Studies.

Table S2. Survey-weighted standardized food-group intake and added fat and sugar consumption across PHDI quartiles in the overall population

Dietary component	Overall, mean (SD)	Q1, mean (SD)	Q2, mean (SD)	Q3, mean (SD)	Q4, mean (SD)	*p*-value	*p* for trend
Whole grains	20.2 (13.8)	12.7 (10.3)	20.5 (14.1)	21.6 (12.6)	25.9 (14.4)	<0.001	<0.001
Vegetables	104.4 (62.7)	71.2 (41.5)	92.5 (53.4)	114.2 (59.8)	139.5 (70.7)	<0.001	<0.001
Fruit	103.6 (76.9)	55.8 (47.1)	93.3 (70.6)	117.8 (75.0)	147.4 (79.5)	<0.001	<0.001
Legumes	6.4 (8.9)	4.4 (5.7)	6.1 (9.0)	6.1 (9.0)	8.9 (10.7)	<0.001	<0.001
Nuts and seeds	8.0 (8.4)	3.5 (3.1)	6.3 (6.2)	7.9 (7.1)	14.4 (11.0)	<0.001	<0.001
Soy	4.6 (24.2)	1.0 (8.7)	4.2 (28.8)	2.5 (13.8)	10.4 (34.6)	<0.001	<0.001
Dairy	133.8 (122.6)	144.8 (158.1)	141.9 (124.0)	133.1 (105.9)	115.5 (89.7)	<0.001	0.002
Fish and shellfish	12.1 (10.3)	7.2 (6.1)	10.7 (9.0)	14.4 (11.5)	15.9 (11.2)	<0.001	<0.001
Poultry	18.7 (15.5)	20.4 (19.0)	19.6 (15.3)	18.5 (14.9)	16.2 (11.5)	<0.001	<0.001
Eggs	11.7 (13.4)	14.6 (19.5)	11.2 (12.1)	10.6 (9.4)	10.4 (9.9)	0.003	<0.001
Red and processed meat	29.8 (17.6)	38.0 (18.7)	33.3 (17.9)	28.7 (14.6)	19.3 (12.9)	<0.001	<0.001
Starchy vegetables	31.9 (22.9)	38.7 (26.9)	35.3 (22.6)	30.7 (21.9)	22.7 (15.4)	<0.001	<0.001
Added unsaturated fat	10.7 (5.2)	8.9 (4.2)	10.2 (4.5)	10.7 (4.4)	13.3 (5.6)	<0.001	<0.001
Added saturated and trans fat	16.9 (5.2)	19.1 (5.7)	17.5 (5.0)	16.5 (4.7)	14.4 (4.4)	<0.001	<0.001
Added sugar	28.5 (17.8)	39.5 (23.9)	29.1 (15.7)	24.8 (12.5)	20.6 (10.2)	<0.001	<0.001

Table S3. Survey-weighted standardized food-group intake and added fat and sugar consumption across PHDI quartiles among men

**Dietary component**	**Overall, mean (SD)**	**Q1, mean (SD)**	**Q2, mean (SD)**	**Q3, mean (SD)**	**Q4, mean (SD)**	***p*-value**	***p* for trend**
Whole grains	19.7 (14.0)	12.0 (9.3)	19.5 (14.1)	22.1 (13.4)	27.3 (14.5)	<0.001	<0.001
Vegetables	90.4 (54.3)	64.2 (35.5)	81.2 (46.4)	102.8 (58.7)	121.5 (58.7)	<0.001	<0.001
Fruit	92.4 (72.4)	49.1 (40.6)	87.0 (73.5)	106.3 (68.2)	139.6 (74.2)	<0.001	<0.001
Legumes	6.0 (8.5)	4.8 (6.5)	5.4 (7.6)	6.7 (10.9)	7.5 (8.6)	0.015	0.001
Nuts and seeds	8.4 (8.5)	3.8 (3.3)	7.3 (7.4)	8.8 (7.6)	15.4 (10.4)	<0.001	<0.001
Soy	4.1 (23.4)	0.9 (9.5)	4.4 (29.6)	3.3 (15.5)	8.9 (32.9)	0.008	0.004
Dairy	124.4 (103.8)	133.4 (115.0)	131.5 (122.6)	122.7 (90.6)	106.3 (71.9)	0.074	0.021
Fish and shellfish	11.8 (9.6)	7.5 (5.9)	11.4 (9.3)	14.0 (9.8)	15.6 (11.2)	<0.001	<0.001
Poultry	17.6 (14.7)	19.8 (17.1)	18.0 (14.3)	16.6 (14.6)	15.3 (11.4)	0.013	0.002
Eggs	12.0 (11.7)	15.2 (15.3)	11.5 (10.5)	10.3 (8.1)	10.3 (9.9)	0.003	<0.001
Red and processed meat	31.3 (17.6)	38.4 (17.9)	35.4 (18.8)	28.3 (14.0)	20.9 (13.1)	<0.001	<0.001
Starchy vegetables	31.4 (22.0)	36.1 (23.7)	33.7 (20.9)	31.6 (23.9)	22.5 (15.4)	<0.001	<0.001
Added unsaturated fat	10.6 (4.7)	8.8 (4.3)	10.2 (4.1)	10.8 (4.5)	13.3 (4.8)	<0.001	<0.001
Added saturated and trans fat	16.8 (5.2)	18.9 (5.5)	17.6 (4.9)	15.6 (4.5)	14.2 (4.1)	<0.001	<0.001
Added sugar	28.8 (17.3)	38.7 (22.3)	27.1 (13.8)	25.6 (13.5)	21.2 (9.6)	<0.001	<0.001

Table S4. Survey-weighted standardized food-group intake and added fat and sugar consumption across PHDI quartiles among women

Dietary component	Overall, mean (SD)	Q1, mean (SD)	Q2, mean (SD)	Q3, mean (SD)	Q4, mean (SD)	p-value	p for trend
Whole grains	20.6 (13.6)	13.5 (11.2)	21.2 (14.0)	21.2 (12.0)	25.1 (14.2)	<0.001	<0.001
Vegetables	115.6 (66.6)	78.6 (46.0)	101.8 (56.9)	122.7 (59.4)	151.4 (75.4)	<0.001	<0.001
Fruit	112.6 (79.2)	63.0 (52.2)	98.4 (67.9)	126.4 (78.7)	152.5 (82.5)	<0.001	<0.001
Legumes	6.7 (9.2)	4.0 (4.6)	6.7 (10.0)	5.7 (7.2)	9.9 (11.8)	<0.001	<0.001
Nuts and seeds	7.7 (8.4)	3.1 (2.9)	5.5 (5.0)	7.3 (6.6)	13.7 (11.3)	<0.001	<0.001
Soy	4.9 (24.8)	1.2 (7.8)	4.0 (28.2)	1.9 (12.5)	11.4 (35.7)	0.001	0.001
Dairy	141.4 (135.4)	156.8 (192.9)	150.4 (124.8)	140.8 (115.5)	121.5 (99.3)	0.008	0.006
Fish and shellfish	12.3 (10.8)	7.0 (6.4)	10.1 (8.7)	14.7 (12.6)	16.1 (11.2)	<0.001	<0.001
Poultry	19.6 (16.0)	21.1 (20.8)	20.8 (15.9)	19.9 (15.0)	16.8 (11.4)	0.002	0.006
Eggs	11.4 (14.7)	13.9 (23.1)	11.0 (13.2)	10.8 (10.4)	10.4 (9.9)	0.279	0.057
Red and processed meat	28.6 (17.5)	37.6 (19.5)	31.6 (17.0)	29.0 (15.0)	18.3 (12.7)	<0.001	<0.001
Starchy vegetables	32.2 (23.6)	41.4 (29.7)	36.6 (23.9)	30.0 (20.4)	22.9 (15.4)	<0.001	<0.001
Added unsaturated fat	10.8 (5.2)	8.9 (4.2)	10.2 (4.8)	10.6 (4.3)	13.2 (6.1)	<0.001	<0.001
Added saturated and trans fat	17.0 (5.3)	19.4 (5.8)	17.4 (5.1)	17.2 (4.7)	14.5 (4.6)	<0.001	<0.001
Added sugar	28.3 (18.3)	40.5 (25.5)	30.7 (16.9)	24.2 (11.7)	20.3 (10.6)	<0.001	<0.001
